# Development of Micellar HPLC-UV Method for Determination of Pharmaceuticals in Water Samples

**DOI:** 10.1155/2018/9143730

**Published:** 2018-03-01

**Authors:** Danielle Cristina da Silva, Cláudio Celestino Oliveira

**Affiliations:** ^1^Universidade Tecnológica Federal do Paraná, Campus Dois Vizinhos, Estrada para Boa Esperança, Km 04 85660-000 Dois Vizinhos, PR, Brazil; ^2^Departamento de Química, Universidade Estadual de Maringá, Avenida Colombo, 5790 87020-900 Maringá, PR, Brazil

## Abstract

Method for extraction and determination of amoxicillin, caffeine, ciprofloxacin, norfloxacin, tetracycline, diclofenac, ibuprofen, nimesulide, levonorgestrel, and 17*α*-ethynylestradiol exploiting micellar liquid chromatography with PDA detector and solid-phase extraction was proposed. The usage of toxic solvents was low; the chromatographic separation of the medicaments was performed using a C18 column and mobile phases A and B containing 15.0% (v/v) ethanol, 3.0% (m/v) sodium dodecyl sulfate (SDS), and 0.02 mol·L^−1^ phosphate at pHs 7.0 and 8.0, respectively. The method is simple, selective, and fast, and the analytes were separated in 23.0 min. For extraction, 1000 mL of sample containing 2.0% (v/v) ethanol and 0.002 mol·L^−1^ citric acid at pH 2.50 was loaded through a 1000 mg of C18 cartridge. The analytes were eluted using 3.0 mL of ethanol, which were evaporated and redissolved in 0.5 mL of mobile phase. Concentration factors better than 1200, except amoxicillin (224), were obtained. The analytical curves were linear (*R* ^2^ better than 0.992); LOD and LOQ (*n*=10) presented values in the range of 0.019–0.247 and 0.058–0.752 mg·L^−1^, respectively. Recoveries of 99% were obtained, and the results are in agreement with those obtained by the comparative methods.

## 1. Introduction

For many years, the analysis of environmental contaminants was done to determine the presence of pesticides, air pollutants, petroleum residues, and many other substances designated as conventional pollutants [1, 2]. Recently, a large number of compounds not regulated by countries legislation have been identified as potential pollutants, which have been designated as emerging contaminants [3]. The pharmaceuticals and their metabolites belong to this new class of pollutants, although their presence in the environment is not new [4, 5].

Many tons of these drugs are produced annually and used in human and veterinary medicine. Generally, the exact amount of pharmaceuticals produced is not published in the literature [6], but it is known that Brazil, United States, France, and Germany belong to the group of the world's largest consumers of these drugs [7]. Thus, the monitoring of these compounds in the environment has gained great interest as they are often found in effluents from wastewater treatment plants and natural waters [4, 8, 9].

In Brazil, the population self-medication is common because part of the population does not have access to adequate medical care and has easy access to medicines due to the high number of drugstores, including those with unethical business practices [8, 10]. After the administration, many pharmaceuticals are transformed into one or more metabolites and excreted in the urine and feces, causing serious problems to the environment [4, 8, 10]. It is known that the rampant use of pharmaceuticals such as antibiotics can make some microorganisms resistant to these drugs as some bacteria have the ability to modify their genetic material [6, 11, 12].

Furthermore, some drugs are used in animal treatment in the rearing of livestock, pigs, and chickens, and the waste generated from these activities has become a major source of environmental contamination due to its use as fertilizer in farmland. Thus, the pharmaceutical compounds are not metabolized and their metabolites can pass into groundwater and eventually to watercourses such as rivers and lakes, affecting aquatic life [6, 8, 12]. Another source of environmental contamination by these drugs is associated with the disposal of waste from pharmaceutical companies and hospitals in landfills that can contaminate underground waters [4, 6].

The presence of hormones in the environment has been indicated as responsible for causing endocrine disturbances in human and animal organisms and endocrine disruptors [13, 14]. There is evidence that reproductive system of certain terrestrial and aquatic organisms is affected by estrogen, resulting in the development of abnormalities and reproductive impairment in exposed organisms, even when these drugs are present at low concentrations [6]. Caffeine is a natural stimulant and the most widely consumed psychoactive drug in the world as it is present in soft drinks, coffee, tea, cocoa, and chocolate and is used concomitantly with various medications as a stimulant [15]. Due to its widespread consumption, caffeine has been used as a potential indicator of anthropogenic pollution of surface water resulted from human activity [16, 17].

Due to the growing concern of the presence of antibiotics, anti-inflammatories and hormones in water intended for public supply a number of methodologies devoted to identify and quantify these compounds in various samples that have been proposed. The most common are those involving separation techniques such as gas and liquid chromatography and capillary electrophoresis coupled with several detectors such as MS, UV-Vis, FID, ECD, and others [18–27] associated with SPE with solvents such as formic acid/water [20], methanol/methyl tertiary-butyl ether [21], *n*-hexane/ethyl acetate/methanol [22], or more modern methods such as ultrasonic-assisted extraction/centrifugation and purification with SPE [18] and the pressurized liquid extraction followed by extract purification using SPE [19].

As it is not an easy task the development of extraction and determination methods to chemical compounds with very different characteristics, few methods permit the determination of the analytes present in this paper in a single run. Thus, environmental researchers and laboratories dedicated to the analysis of drugs should use several methods for the determination of drugs from different classes in environmental samples, which increase the cost and time of the analysis. The exception is the method based on MS detector that can furnish adequate results to water analysis of emergent pollutants, but it should be considered that a lot of laboratories dedicated to routine water analysis did not have mass spectrometer, mainly due to the cost to acquire the equipment or lack of skilled professionals capable of properly using the equipment.

Thus, to fill this gap, in the present work is proposed a robust, simple, and green HPLC-UV method associated with SPE extraction for the determination of antibiotics (amoxicillin, ciprofloxacin, norfloxacin, and tetracycline), anti-inflammatories (diclofenac, ibuprofen, and nimesulide), hormones (17*α*-ethynylestradiol and levonorgestrel), and caffeine in water samples, providing the laboratories dedicated to water analysis with a tool able to determine the more representative pharmaceuticals from classes of antibiotics, anti-inflammatories, and hormones used in Brazil in a single chromatographic run and using a simple HPLC-UV; a common equipment in a number of laboratories devoted to contaminant analysis, contributing to environmental researchers involving emerging pollutants. The task can be done exploiting the micellar chromatography as the surfactant can modify the C18 and the aqueous phase, increasing the possibilities of interactions of the analytes with both phases, permitting the separation of substances with different polarities in the same chromatographic run. The selected compounds are considered toxic, are among the most used in Brazil, present biological activity, and their presence in the aquatic environment has already been demonstrated, which justify their determination.

## 2. Experimental

### 2.1. Reagents

All reagents and solvents were of analytical grade (purity higher than 98%); the aqueous solutions were prepared using deionized water and were ultrasonically degassed and vacuum-filtered through a cellulose acetate membrane of 0.45 *μ*m before chromatographic use.

Solutions containing ethanol or *n*-propanol or *n*-butanol of HPLC-grade (Tedia, Fairfield, USA), anhydrous dibasic sodium phosphate (Na_2_HPO_4_), SDS, or CTAB (cetyl trimethyl ammonium bromide), or SDBS (sodium dodecyl benzene sulfonate), or Triton X-100 (polyoxyethylene (9-10) *p*-phenol tertoctyl) (both from Sigma-Aldrich, St. Louis, USA) at different concentrations and pH values were tested as mobile phase. The main mobile phases containing 3.0% (m/v) SDS, 20.0 mmol·L^−1^ Na_2_HPO_4_, and 15.0% (v/v) ethanol at pH 7.0 (phase A) and pH 8.0 (phase B) were prepared by dissolving 3.046 g of SDS, 0.284 g of Na_2_HPO_4_, and 15.0 mL of ethanol at approximately 70.0 mL of water and leaving the solution under stirring until complete homogenization. Later, the pH was adjusted by adding HCl or NaOH 1.0 mol·L^−1^, and the final volume was made up to 100.0 mL in a volumetric flask. All mobile phases used in this work were prepared following the same procedures and just modifying the percentages of reagents or changing the organic solvent to *n*-propanol or *n*-butanol or the surfactant.

Amoxicillin, ciprofloxacin, norfloxacin, tetracycline, diclofenac, ibuprofen, nimesulide, 17*α*-ethynylestradiol, levonorgestrel, and caffeine were supplied by Sigma-Aldrich (St. Louis, USA), and 20.0 mg·L^−1^ stock solutions were prepared by dissolving 1.0 mg of each drug in the mobile phase A, the solution was submitted to sonication for 3.0 min to ensure complete dissolution of analytes, and the volume was completed to 50.0 mL in a volumetric flask. The solutions were stored in amber flasks, protected from light, and were frozen. Just before analysis, the solutions were left to attain thermal equilibrium with the room temperature.

To verify the stability of each drug in the mobile phase and the wavelength to monitor them, 50 mL of 10.0 mg·L^−1^ of each drug standard solution was prepared in the mobile phase A and divided into two portions of 25.0 mL. The first set was frozen and protected from light, and the second set was kept at 25°C and immediately used to obtain the UV-Vis spectra (200 to 600 nm). The procedure was repeated each 60 min for 3 h during the first day and then the solution was stored in the refrigerator at 10°C. Later, the solutions (two sets) were analyzed every day during 4 days.

### 2.2. Sampling, Sample Treatments, and Optimization of the SPE Procedure

A total of 7 river water samples were collected from rivers flowing in the center of Maringá (Paraná, Brazil) in zones with different population densities and industrial activities. Samples 1–3 were collected from the south of Maringá in Moscados stream (ca. 2.9 km from its source in the Inga Park), Cleópatra stream (ca. 2.3 km from its source, located inside the Pioneiros Forest Park), and Borba Gato stream (ca. of 3.3 km from its source located in the Horto Florestal Park), respectively. Samples 4–6 were collected in the north of Maringá in Mandacarú stream (ca. 2.0 km from the source), Morangueiro stream (ca. 4.0 km from the source), and Maringá stream (ca. 2.0 km from the search), respectively; sample 7 was tap water collected in the analytical chemistry laboratory at Maringá State University.

Before sample collection, each bottle was prerinsed with the sample for three times. The samples were sent in boxes packed with ice to the laboratory at Maringá State University. Immediately upon reception, the samples were vacuum filtered through Millipore 0.45 *µ*m membrane; then, to 1.0 L of each sample was added 2.0% (v/v) ethanol and 2.0 mmol·L^−1^ citric acid, and the pH was adjusted to 2.5 with HCl 1.0 mol·L^−1^.

For sampling, 4 L of each water sample was collected according to the standard protocol established by the Water Resources Company Management [28], which considers sampling timing, sampling point, sampling tools and containers, sampling operation, field records, labeling, transport, and storage of samples. Immediately upon reception, the samples were vacuum filtered through Millipore 0.45 *μ*m membrane and to 1.0 L of each sample was added 2.0% (v/v) ethanol and 2.0 mmol·L^−1^ citric acid, and the pH was adjusted to 2.5 with HCl 1.0 mol·L^−1^. Later, the samples were submitted to the following extraction procedure: before the extraction, the C18 stationary phase was preconditioned by passing 5 mL of methanol, 5 mL of pure water, and 5 mL of an aqueous solution containing 2.0% (v/v) ethanol and 2.0 mmol·L^−1^ citric acid at pH 2.5; then, 1000 mL of each sample was passed through the cartridge at a flow rate of 7.0 mL·min^−1^. The solid phase was dried under vacuum for 30 s, and the analytes were eluted using 3.0 mL of ethanol at 1.0 mL·min^−1^. The sample was filtered through a teflon membrane, evaporated, and redissolved in 0.5 mL with the mobile phase A and injected into the chromatograph. To obtain the extraction procedure able to be applied to the ten analytes, the following variables were tested: pH (2.0–8.7), sample (1.0–10.0 mL·min^−1^) and eluent (0.5–3.0 mL·min^−1^) flow rates, sample (25.0–1000 mL) and eluent (1.0–10 mL) volumes, amount of stationary phase (500 and 1000 mg), and chemical nature of the eluent (methanol, ethanol, acetone, and acetonitrile).

### 2.3. Equipment and Separation Conditions

The chromatographic separation was performed using an HPLC from Thermo Electron Corporation (Waltham, USA) containing quaternary pump model Surveyor LC Plus, manual injector valve of 20 *μ*L Rheodyne Model 8096, UV-Vis photodiode array detector model Surveyor PDA with quartz cell with optical path of 5.0 cm, ChromQuest software version 4.2 (Macherey-Nagel, Germany) for acquisition and signal recording and a column RP-18 ODS off base 250 × 4.6 mm (id) equipped with a guard column RP-18 ODS 10 × 4.0 mm (id) both with particles of 5-micron pore size of 100 Å and carbon content of 15.5%.

Before the first and after the last injection of the day, the column was cleaned with ultrapure water for 30.0 min at a flow rate of 0.5 mL·min^−1^. The initial conditioning of the stationary phase was performed by passing mobile phase A through the column for 20.0 min at a flow rate of 1.0 mL·min^−1^. After standard/sample injection (20.0 *μ*L), the separation process was carried out as follows: 0.0–2.0 min with 100.0% of phase A with the instantaneous change to the 100.0% of phase B and keeping it until 25 min always at a flow rate of 1.0 mL·min^−1^. The temperature was fixed at 25°C, and the analytes were monitored at 220 (amoxicillin, norfloxacin, tetracycline, diclofenac, ibuprofen, nimesulide, 17*α*-ethynylestradiol and caffeine), 240 (levonorgestrel), and 280 nm (ciprofloxacin), simultaneously. After each analysis, the column was reconditioned for 10.0 min using phase A at a flow rate of 1.0 mL·min^−1^.

### 2.4. Optimization of the Chromatographic Method

Preliminary experiment was done to identify the main variables affecting the chromatographic separation such as pH (5.0, 6.0, 7.0, and 8.0), percentage of the organic modifier in the mobile phase (15.0, 20.0, and 25.0% (v/v)), nature of the organic modifier (*n*-butanol, *n*-propanol, and ethanol), SDS concentration (2.5, 3.0, and 3.5% (m/v)), and flow rate (0.8, 1.0, 1.2, and 1.5 mL·min^−1^). Therefore, a factorial design experiment 2^3^ was carried out in duplicate in order to optimize the separation conditions and the contribution of the variables (Na_2_PO_4_, ethanol, and SDS concentrations) in the chromatographic separation (Table 1).

### 2.5. Method Calibration, Characterization, and Sample Analysis

For quantitative determination of pharmaceuticals and caffeine in the water samples, calibration curves were plotted using the peak area (*y*) versus concentration of the analytes in the following ranges: amoxicillin, ciprofloxacin, diclofenac, ibuprofen, tetracycline, levonorgestrel, nimesulide, and norfloxacin from 0.1 up to 25.0 mg·L^−1^; 17*α*-ethynylestradiol from 0.5 up to 25.0 mg·L^−1^; and caffeine from 0.08 up to 25.0 mg·L^−1^. The calibration curves were used to determine the analyte concentrations in the samples and in the blank with and without spiking.

The LOD and LOQ (*n*=10) were estimated using the signal-to-noise ratio of 3.3 and 10.0, respectively [9], and 7 river water samples were analyzed by the proposed method and the obtained results were compared to those obtained by the six comparative HPLC-DAD methods involving SPE [18, 24, 29, 30], stir-bar sorptive and liquid desorption [25], and liquid-liquid extraction [31] methods to the following analytes: tetracycline [18]; ibuprofen, diclofenac, and nimesulide [24]; levonorgestrel and 17*α*-ethynylestradiol [25]; caffeine [29]; ciprofloxacin and norfloxacin [30]; and amoxicillin [31].

Sample recovery tests were done spiking the samples with the following: 5.0 *µ*g·L^−1^ of ibuprofen, 17*α*-ethynylestradiol, diclofenac, nimesulide, levonorgestrel, ciprofloxacin, and norfloxacin; 10 *µ*g·L^−1^ of tetracycline and caffeine; and 60 *µ*g·L^−1^ of amoxicillin. After extraction procedure, the extracts were evaporated completely and stored in the refrigerator; just before analysis, the samples were redissolved in 0.5 mL of the mobile phase A. The spiked samples were analyzed (*n*=5) by the proposed and by comparative methods during five consecutive days (*n*=5).

## 3. Results and Discussion

### 3.1. Spectra and Stability of the Analytes

The UV-Vis spectra of analytes showed electromagnetic radiation absorption in the region between 200 and 600 nm with higher intensity in the UV region. It is observed that signal overlaps, which makes it difficult for the simultaneous determination of the analytes without prior separation. It was decided to monitor the analytical signals at 220, 240, and 280 nm, because the molar absorptivity is high to the majority of the analytes and the mobile phase absorption is low; the exceptions are levonorgestrel (*λ*_max_ = 240 nm) and ciprofloxacin (*λ*_max_ = 280 nm) that presented low molar absorptivity at 220 nm. For this reason, the signals can be monitored at 220 nm by laboratories that do not have chromatograph with PDA detector, but the sensitivity to levonorgestrel and ciprofloxacin will be poor.

The UV-Vis spectra also indicated that the analytes were stable in the mobile phase A for a long period, except tetracycline that presented degradation of 4.1, 8.5, 13.4, and 16.0% after 24, 48, 72, and 96 h, respectively. When the samples were kept frozen and in the absence of light before analysis, the tetracycline degradation decreased to lower than 2.0%, indicating that the standards and samples should be kept under these conditions until analysis.

### 3.2. Extraction of the Analytes

Initially, it was decided to use the C18 phase, control the pH, and use methanol as eluent, as 10 mL of this solvent permitted to get the elution of ten analytes from the cartridge.

The sample pH was varied from 2.0 up to 8.5, and pH 2.5 was chosen as the better condition due to the optimum extraction percentage for most of the analytes. Amoxicillin, high polar molecule, presented low affinity by the solid phase, and its recuperation was always lower than 51%, whereas ibuprofen, diclofenac, and nimesulide presented high interaction with the solid phase and could only be eluted around pH 2.0 with recoveries of 83.6, 59.0, and 75.5%, respectively. Ciprofloxacin, norfloxacin, and tetracycline have two positive charges at pH 2.5, and the increase in the pH reduced their recuperation from 92.0, 89.0, and 100.0% for pH 2.5 to 36.3, 19.4, and 42.2% for pH 8.5, respectively; and caffeine, 17*α*-ethynylestradiol, and levonorgestrel did not suffer a significant change in their interaction with the solid phase with the pH and presented recuperation percentages of 94.7, 92.0, and 80.0%, respectively.

A flow rate of 7.0 mL·min^−1^ was chosen as the better compromise between time and efficiency of extraction; for this condition, recoveries around 95% were obtained for the analytes; the exception was amoxicillin (50%). Variations in the citric acid concentration (2.0, 3.0, and 5.0 mmol·L^−1^) did not change the extraction efficiencies; thus, 2.0 mmol·L^−1^ of citric acid was maintained.

The loading sample volume should be as high as possible to get high concentration factors. When solutions of the analytes in the concentrations of 1.0, 0.5, 0.25, 0.05, and 0.025 mg·L^−1^ were associated with extraction sample volumes of 25, 50, 100, 500, and 1000 mL (to keeping the final concentration after elution in 2.5 mg·L^−1^), it was noted an improvement in the extraction with the increase of sample volume; however, for the more polar analytes (amoxicillin, tetracycline, caffeine, and diclofenac), a reduction in the recovery percentages was observed. For sample volume of 500 mL, the best recoveries for most analytes were obtained with values higher than 85%, except for amoxicillin (15.3%); however, in order to obtain high concentration factor to levonorgestrel and 17*α*-ethynylestradiol hormones, that could be present at very low concentrations in the samples, the condition of 1000 mL was selected, even with a reduction in the extraction efficiency to amoxicillin, tetracycline, caffeine, and diclofenac.

The amount of solid phase in the cartridge was also studied, and it was noted that reducing the amount of solid phase led to lower extraction efficiency to tetracycline and amoxicillin. Thus, the cartridge with 1000 mg of C18 was selected.

In order to achieve the highest possible concentration factor with minimum use of organic solvents (methanol, ethanol, acetone, and acetonitrile), the volume of the solvent was varied (1.5, 3.0, 5.0, 7.0, and 10.0 mL) and it was not observed significant variations in the analytes recoveries when the eluent was methanol or ethanol and the eluent volume was varied from 3.0 to 10.0 mL; thus, 3.0 mL of ethanol was elected as the better condition due to its low toxicity. To acetone and acetonitrile, the recoveries were low to all analytes, mainly to tetracycline, norfloxacin, and ethynylestradiol. The eluent flow rate was varied, and it was observed a decrease in the extraction efficiencies for all the analytes to flow rates higher than 1.0 mL·min^−1^; then, the eluent flow rate was fixed at 1.0 mL·min^−1^.

Thus, the final extraction conditions for simultaneous extraction and concentration of the ten analytes were as follows: 1000 mL of the sample containing 2.0% (v/v) ethanol, 2.0 mmol·L^−1^ citric acid at pH 2.50, C18 cartridge with 1000 mg of the solid phase, and flow rate of 7.0 mL·min^−1^. After analytes' retention, the adsorbent was dried under vacuum 30 s and the analytes were eluted with only 3.0 mL of ethanol at a flow rate of 1.0 mL·min^−1^. The solvent was evaporated, and the samples were redissolved in 0.5 mL of a solution with 15.0% (v/v) ethanol, 3.0% (m/v) SDS, and 20.0 mmol·L^−1^ phosphate buffer at pH 7.0 (mobile phase A). Under these conditions, it was yielded recovery percentage values of higher than 95%, except to tetracycline (64.3%) and caffeine (66.0%), and concentration factors higher than 1200, except to amoxicillin (224).

### 3.3. Chromatographic Separation

#### 3.3.1. Preliminary Tests

The developed chromatographic method should be as green as possible, and taking into account the different polarities of the analytes, it was decided to exploit the micellar chromatography [32, 33] due to the possibility to get the solubilization of organic compounds of low polarity in the aqueous medium and, at the same time, change the polarity of the C18 stationary phase with the surfactants. For the task, SDS, CTAB, SDBS, and Triton X-100 surfactants were tested and the anionic surfactant SDS was chosen because of its low critical micellar concentration (cmc of 0.0082 mol·L^−1^ in water), which permits to use lower surfactant concentration. Furthermore, SDS presents low light absorption and scattering effect in the UV region, where the analytes should be monitored, allowing to get lower baseline signals and better sensitivity.

**Figure 1:**
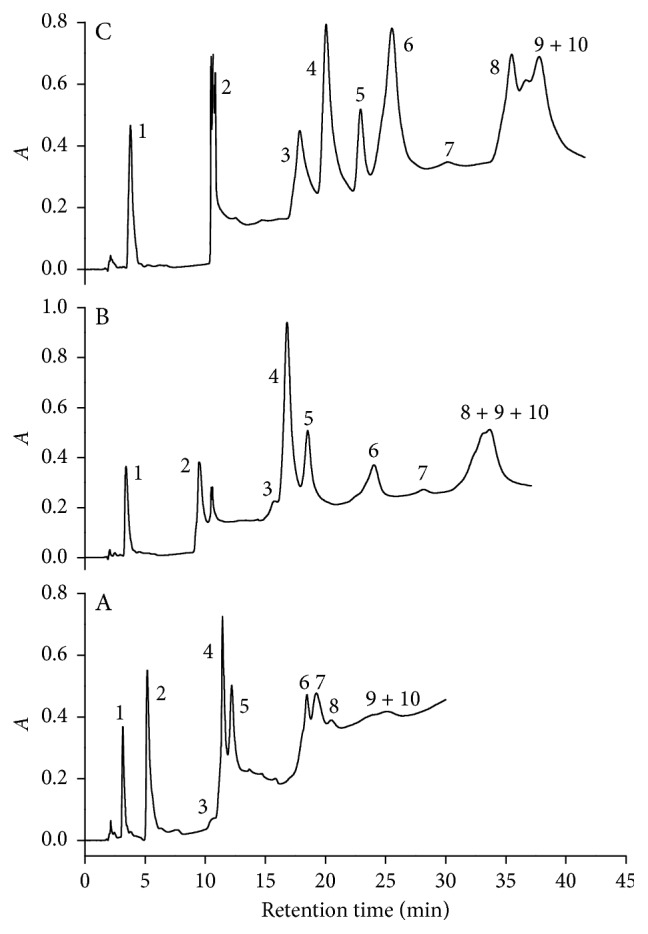
Influence of the nature of the organic modifier in the analyte separation. Solvents: (a) *n*-butanol, (b) *n*-propanol, and (c) ethanol. Data were obtained for standard solution of amoxicillin (1), caffeine (2), tetracycline (3), diclofenac (4), ibuprofen (5), 17*α*-ethynylestradiol (6), levonorgestrel (7), nimesulide (8), ciprofloxacin (9), and norfloxacin (10) in the concentration of 20.0 mg·L^−1^, with a injected volume of 20 *μ*L, at a flow rate of 1.0 mL·min^−1^, at 25°C, and detection at 220 nm. Phase A with 3.0% (v/v) of solvent and 0.3% (m/v) SDS and phase B with 15% (v/v) of solvent and 3.0% (m/v) SDS and both with 20 mmol·L^−1^ of phosphate at pH 7.0. The mobile phase was changed from 100% of phase A to 100% of phase B in 30 min. *A* in the *y*-axis is the absorbance.

Low toxic organic modifier solvent to assist the solubilization of the ten analytes in the micellar medium was chosen after solubility tests with several proportions of ethanol or *n*-propanol or *n*-butanol with water and SDS. It was observed that solutions containing 15% of ethanol or butanol or *n*-propanol together with at least 3.0% SDS and 20 mmol·L^−1^ of phosphate buffer at pH 7.0 were able to solubilize all the analytes as well as elute them from the C18 column (Figure 1). The differences in the chromatograms for the different organic modifiers can be attributed to the different polarities of each organic solvent aliphatic chain, which explains the highest retention time obtained when ethanol was used as an organic modifier (Figure 1). Due to this effect, ethanol was considered the better organic modifier because it improved the symmetry and chromatographic resolution of some peaks (except nimesulide, ciprofloxacin, and norfloxacin) and is low toxic than *n*-butanol and *n*-propanol. In addition, the use of ethanol permitted the identification of the tetracycline peak, which could not be done when it was used *n*-propanol or *n*-butanol.

#### 3.3.2. pH Effect in the Chromatographic Separation

**Figure 2:**
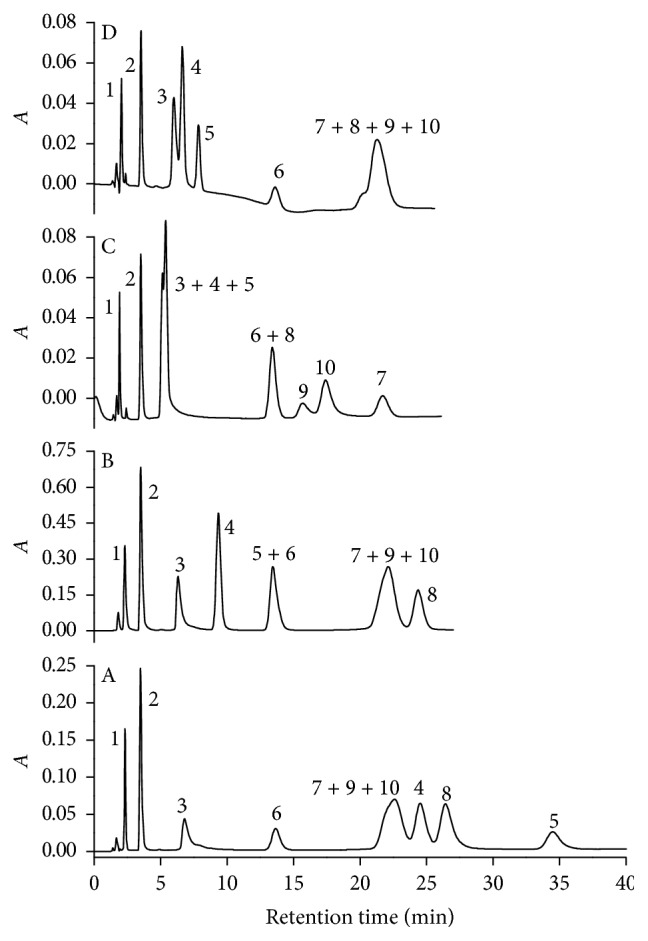
Effect of pH on the analyte separation. pH: (a) 6.0; (b) 7.0; (c) 8.0, and (d) 50% of phase A (pH 7.0) and 50% of phase B (pH 8.0). Data were obtained for standard solution of amoxicillin (1), caffeine (2), tetracycline (3), diclofenac (4), ibuprofen (5), 17*α*-ethynylestradiol (6), levonorgestrel (7), nimesulide (8), ciprofloxacin (9), and norfloxacin (10) in the concentration of 20.0 mg·L^−1^, with an injected volume of 20 *μ*L, at a flow rate of 1.0 mL·min^−1^, at 25°C, and detection at 220 nm. Mobile phase contains 15.0% (v/v/) ethanol, 3.0% (m/v) SDS, and 20.0 mmol·L^−1^ of phosphate and isocratic elution. *A* in the *y*-axis is the absorbance.

pH is an important variable to be studied when the analytes have ionizable groups, as variations in pH can promote changes in the solubility and in the ionic interactions of analytes with the micellar medium and with the stationary phase. When the pH was varied from 6.0 up to 8.0 (Figure 2), caffeine (peak 2, p*K*a 2.19), 17*α*-ethynylestradiol (peak 6, p*K*a 9.44), and levonorgestrel (peak 7, p*K*a  1.05) did not show significant variation in their retention time as these compounds are neutral in that pH range; the different retention time periods were attributed to their different hydrophobicities. On the other hand, the density of negative charges increased with pH to diclofenac (peak 4, p*K*a 5.35), ibuprofen (peak 5, p*K*a 5.82), and nimesulide (peak 8, p*K*a 7.15), causing large reduction in their retention time due to the high affinity of the charged analytes by the micellar aqueous mobile phase and their repulsion by the negatively charged groups of the surfactants adsorbed on the stationary phase.

Tetracycline (p*K*a's 3.94, 7.62, and 9.19), ciprofloxacin (p*K*a's 3.32, 7.12, and 8.42), and norfloxacin (p*K*a's 3.38, 7.16, and 8.45) have positive charge densities in the tested pH interval and they are the most soluble molecules in aqueous medium, but their electrostatic interaction with the negative part of the surfactant chain attached to C18 phase increased their retention time to values to those obtained with the hydrophobic compounds. The effect also caused peak broadening, and at pH 5.0, it was not possible to identify the tetracycline peak due to excessive molecule retention. Amoxicillin (p*K*a's 3.01, 7.32, and 9.70, pI 5.20) is also an amphoteric molecule and with the pH reduction from 7.0 to 6.0 (Figures 2 and 2), its retention time increased because the molecule almost reached the neutrality and became more hydrophobic.

In none of the tested pH conditions was possible to obtain the chromatographic separation of the ten analytes with resolutions adequate to their analytical determination. Due to the different analyte characteristics, they could be divided into two groups: those with symmetric and thin peaks and presenting low retention time and those presenting broad peaks, high retention time, and low efficiency and chromatographic resolution.

Then, it was decided to do a mobile phase pH gradient starting with 100% of phase A changing gradually (7.0, 5.0, and 2.0 min) to 100% of phase B (same composition of phase A at pH 8.0). The phase with pH 6.0 was not chosen because some analytes were highly retained in the stationary phase under this condition (Figure 2).

**Figure 3:**
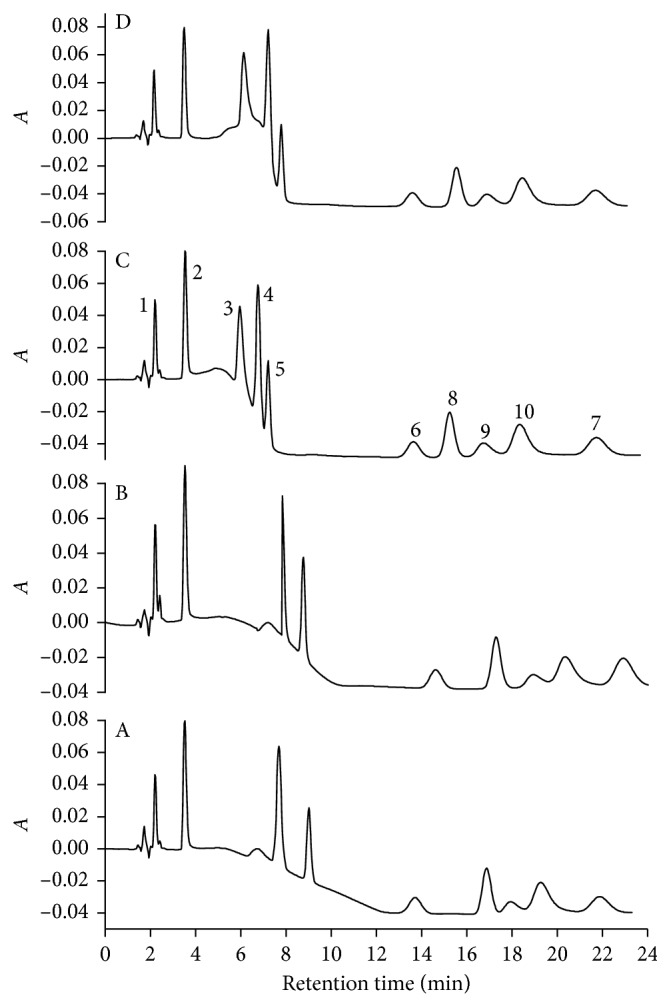
Effect of the pH gradient on the analyte separation. Gradients: (a) 7.0 min, (b) 5.0 min, (c) 2.0 min, and (d) isocratic until 2.0 min. Data were obtained for standard solution of amoxicillin (1), caffeine (2), tetracycline (3), diclofenac (4), ibuprofen (5), 17*α*-ethynylestradiol (6), levonorgestrel (7), nimesulide (8), ciprofloxacin (9), and norfloxacin (10) in the concentration of 20.0 mg·L^−1^, with an injected volume of 20 *μ*L, at a flow rate of 1.0 mL·min^−1^, at 25°C, and detection at 220 nm. Mobile phase contains 15.0% (v/v/) ethanol, 3.0% (m/v) SDS, and 20.0 mmol·L^−1^ of phosphate at pH 7.0 (phase A) and pH 8.0 (phase B). *A* in the *y*-axis is the absorbance.

It was observed that as sooner B phase was introduced, lower analytes' retention time and better analytes separation were obtained, especially to nimesulide (peak 8), ciprofloxacin (peak 9), norfloxacin (peak 10), and levonorgestrel (peak 7) (Figure 3). The chromatographic resolution between diclofenac (peak 4) and ibuprofen (5) worsened, but without compromising their quantification. Thus, it was concluded that the better pH gradient would be 100% of phase A until 2.0 min with a sudden change to 100% of phase B 100%. Under this condition, it was possible to get the separation of the ten compounds with adequate chromatographic resolution in only 23.0 min (Figure 3).

#### 3.3.3. Variables' Interactions

The preliminary experiments demonstrated that the use of ethanol as an organic modifier and gradient of phases A and B were adequate to achieve the analyte separation; thus, a factorial design 2^3^ (Table 1) with SDS, ethanol, and phosphate concentrations as variables in both phases was carried out to verify the main and the interaction effects of variables and their importance in the analytes separation.

**Figure 4:**
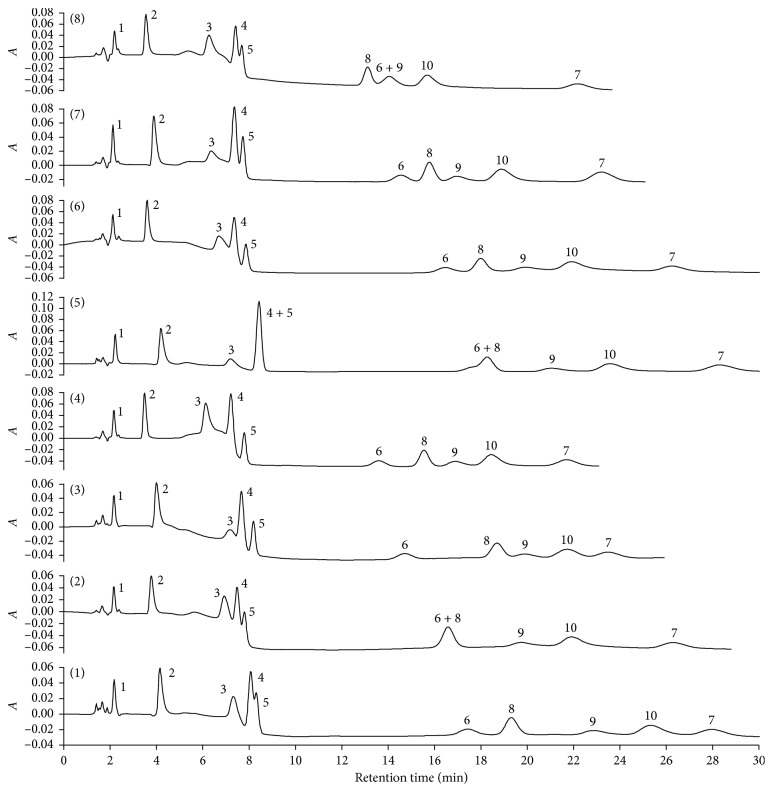
Chromatograms obtained from factorial design 2^3^. Numbers from (1) to (8) correspond to the experimental condition number. *A* in the *y*-axis is the absorbance.

Reducing ethanol concentration in the mobile phase from 15.0% to 12.0% (v/v) promotes an increase in the analyte retention time and in time of analysis (experiments 4 and 3, Figure 4). The effect can be explained by the reduction of nonpolar characteristic of the mobile phase, which is difficult for the elution of the low polar analytes from the C18 phase, which led to an overlap between tetracycline (peak 3) and diclofenac (peak 4), nimesulide (peak 8) and ciprofloxacin (peak 9), and norfloxacin (peak 10) and levonorgestrel (peak 7), decreasing their chromatographic resolutions (Table 2).

The reduction in SDS concentration from 3.0% to 2.5% (m/v), experiments 4 and 2 (Figure 4), also increased the retention time for peaks 3, 4, 6, 9, 10, and 7. The reduction in the number of micelles in the mobile phase increased the hydrophobic interaction of 17*α*-ethynylestradiol (peak 6) and levonorgestrel (peak 7) by C18 phase, causing the coelution of 17*α*-ethynylestradiol (peak 6) with nimesulide (peak 8). The compounds 3, 4, 9, 10, and 7 increased their affinities by the stationary phase due to electrostatic interactions between analytes, partially positive charged, and the negative ionized sulfonic acid groups adsorbed on C18 phase. This factor improved the separation between nimesulide (peak 8) and ciprofloxacin (peak 9) and ibuprofen (peak 9) and norfloxacin (peak 10) (Table 2).

Analyzing the results furnished by experiments 4 and 8, it was noted that the increase in the phosphate concentration (from 20 to 30 mmol·L^−1^), and therefore Na^+^ concentration, reduced the affinity of ibuprofen (peak 5), nimesulide (peak 8), norfloxacin (peak 10), and ciprofloxacin (peak 9) by the stationary phase due to two reasons. First, because ion-exchange competition between Na^+^ and the positively charged analytes by the ionized sulfonic acid groups absorbed on C18 phase and, second, due to the partial stabilization of the analytes charged by the phosphate ions. Thus, the chromatographic resolutions between diclofenac (peak 4) and ibuprofen (peak 5) and 17*α*-ethynylestradiol (peak 6) and nimesulide (peak 8) decreased from 1.68 and 2.10 to 0.51 and 1.42, respectively (Table 2). The variations in ethanol and SDS concentration practically did not affect the amoxicillin peak, whereas caffeine experienced a slight increase in its retention time when ethanol concentration was decreased.

The association of lower percentages of SDS and ethanol led to higher time of analysis (30 min), and despite an improvement in the resolution between peaks 6 and 10, the resolution between diclofenac (peak 4) and ibuprofen (peak 5) decreased (Figure 4, experiment 1, Table 2). Furthermore, when these factors were associated with higher phosphate concentrations (Figure 4, experiment 5), it was observed the coelution of diclofenac (peak 4) and ibuprofen (peak 5) and 17*α*-ethynylestradiol (peak 6) and nimesulide (peak 8).

From the main effects (Table 3), it was possible to note that ethanol factor improved the chromatographic resolution between the analytes, especially between peaks 4 and 5, the exception was between peaks 6 and 8. The SDS factor improved the resolution between peaks 6 and 8 and worsened it to peaks 8 and 9. The phosphate factor reduced the resolution between peaks 6 and 8 and increased it to peaks 3 and 4. SDS promoted the higher increase in the time of analysis followed by ethanol, whereas the phosphate concentration presented a contrary effect and induced the reduction in the time of analysis.

The second-order (AB, AC, and BC) and third-order (ABC) interaction effects indicated intense variable interactions (Table 3), showing that the variables could not be studied in an independent form. The ethanol-SDS interaction was significant to increase the resolution between peaks 3 and 4 that was not critical but decreased the chromatographic resolution between peaks 4, 5, 6, and 8 and increased the time of analysis. The ethanol-phosphate interaction was important to increase the resolution between peaks 6 and 8 and contributed to decreasing the resolution between peaks 3 and 4 and 8 and 9. The SDS-phosphate interaction increased the resolution between peaks 8 and 9 and decreased the resolution between peaks 4 and 5 and 6 and 8. The ethanol-SDS-phosphate interaction increased the time of analysis as well as the resolution between peaks 8 and 9 but decreased the resolution between peaks 4 and 5 and 6 and 8 (Table 3).

Considering all variable effects, it was concluded that experiment 4 presented the best characteristics; under this condition, it was possible to separate the ten analytes in only 23.0 min always with chromatographic resolution better than 1.45.

The effect of the flow rate in the analyte separation showed that, for flow rate values higher than 1.0 mL·min^−1^, the chromatographic resolutions worsened, and the separation between tetracycline and diclofenac, diclofenac and ibuprofen, and ciprofloxacin and norfloxacin was poor, then 1.0 mL·min^−1^ was selected. Thus, the final separation conditions were as follows: mobile phase with 15.0% (v/v) ethanol, 3.0% (m/v) SDS, 20.0 mmol·L^−1^ phosphate buffer at pH 7.0 to phase A and 8.0 to phase B; maintaining 100% of phase A until 2.0 min with abrupt change to 100% of phase B; flow rate of 1.0 mL·min^−1^; 25°C; injected volume of 20 *μ*L; monitoring the signals at 220, 240, and 280 nm; and time of analysis of 23.0 min (Figure 4, experiment 4).

### 3.4. Calibration, Characterization, and Sample Analysis

The analytical curves for the ten analytes in the concentration range of 0.10 up to 25.0 mg·L^−1^ for amoxicillin, diclofenac, ibuprofen, levonorgestrel, nimesulide, tetracycline, ciprofloxacin, and norfloxacin; 0.5 up to 25.0 mg·L^−1^ for 17*α*-ethynylestradiol; and 0.08 up to 25.0 mg·L^−1^ for caffeine were linear always with *R* ^2^ better than 0.992. The limits of detection (LOD) and quantification (LOQ), *n*=10, considering the signal/noise ratio of 3.30 and 10.0 times, respectively, were estimated between 0.019 and 0.247 mg·L^−1^ and 0.058 and 0.752 mg·L^−1^ for caffeine and 17*α*-ethynylestradiol, respectively.

When the proposed method was applied to the analysis of the 7 water samples, the standard deviation was ca. 2% and the peaks showed to be free of interferences. The results furnished by the proposed method are in agreement with those obtained by the comparative chromatographic methods (Table 4). Furthermore, the obtained results to sample recovery tests were consistent with high recovery values and low standard deviations (Table 5).

Considering all the obtained results, the proposed method presented analytes average recovery of 99.12% and intraday (*n*=3) and interday (*n*=5) precision of 1.11% and 2.30%, respectively, whereas the comparative methods presented analytes average recovery of 98.64% and intraday (*n*=3) and interday (*n*=5) precision of 1.34% and 2.97%, respectively, indicating that the proposed method furnished results similar to those obtained by the comparative methods, but with the difference that it was necessary to carry out more than one method for the determination of the ten analytes.

The analysis of river water samples indicated the presence of caffeine in all the samples with concentrations ranging from 0.071 mg·L^−1^ to 1.204 mg·L^−1^, probably due to the human activities. It was verified the presence of antibiotics in three streams: tetracycline and norfloxacin in Cleópatra stream, norfloxacin in the Mandacarú stream, and ciprofloxacin in the Morangueiro stream (Table 4).

The proposed chromatographic and extraction method presented high accuracy and precision for the analysis of water samples, allowing the quantification of the ten analytes with only one extraction and chromatographic method.

## 4. Conclusions

The proposed extraction and determination method demonstrated to be able to do the extraction and determination of tetracycline, ibuprofen, 17*α*-ethynylestradiol, caffeine, diclofenac, nimesulide, levonorgestrel, ciprofloxacin, norfloxacin, and amoxicillin using a green mobile phase (15.0% (v/v) ethanol, 3.0% (m/v) SDS, and 20.0 mmol·L^−1^ of phosphate at pHs 7.0 and 8.0) and to extract the analytes in only one procedure using a C18 cartridge and ethanol as eluent. The method was selective, robust, simple, fast, and inexpensive, presented adequate LOD and LOQ, and it can be an important tool for laboratories dedicated to the analysis of emerging pollutants that do not have MS detector.

## Figures and Tables

**Table 1:** Factorial experiment 2^3^ to optimize the separation of the analytes.

Experiment	[Ethanol], % (m/v)	[SDS], % (m/v)	[Phosphate], mmol·L^−1^
1	12	2.5	20
2	15	2.5	20
3	12	3.0	20
4	15	3.0	20
5	12	2.5	30
6	15	2.5	30
7	12	3.0	30
8	15	3.0	30

**Table 2:** Chromatographic resolutions obtained in the factorial design.

Factorial design	Rs (1-2)	Rs (2-3)	Rs (3-4)	Rs (4-5)	Rs (5-6)	Rs (6-8)	Rs (8-9)	Rs (9-10)	Rs (10-7)
1	7.17	5.98	1.27	0.53	9.89	1.62	2.75	1.37	1.58
2	6.39	6.60	0.96	0.98	14.11	0.00	3.42	1.40	2.83
3	6.77	5.98	0.85	1.59	9.07	3.66	1.30	1.06	1.24
**4**	**6.64**	**6.58**	**2.18**	**1.69**	**8.70**	**2.10**	**1.55**	**1.45**	**2.33**
5	7.04	5.33	1.99	0.00	13.44	0.00	2.01	1.42	2.49
6	6.90	5.61	1.06	1.36	10.08	1.30	1.41	1.23	2.81
7	6.99	4.89	1.85	1.08	9.48	1.23	1.26	1.33	2.76
8	5.78	6.26	2.49	0.51	13.53	0.92	1.22	1.47	5.16

Amoxicillin (1), caffeine (2), tetracycline (3), diclofenac (4), ibuprofen (5), 17*α*-ethynylestradiol (6), levonorgestrel (7), nimesulide (8), ciprofloxacin (9), and norfloxacin (10).

**Table 3:** Main effects, interaction among the variables, and time of analysis.

Factor	Effects					
	Rs (3-4)	Rs (4-5)	Rs (6–8)	Rs (8-9)	Rs (9-10)	Time of analysis
A-ethanol	0.190	0.310	−0.580	0.055	0.100	1.730
B-SDS	0.530	0.460	1.150	−1.070	−0.030	4.630
C-phosphate	0.520	−0.410	−0.950	−0.790	0.037	−0.170
AB	0.800	−0.520	−0.390	0.043	0.170	0.280
AC	−0.320	0.026	0.950	−0.390	−0.120	0.075
BC	0.096	−0.420	−0.820	0.590	0.110	−0.025
ABC	−0.029	−0.370	−0.320	0.240	−0.023	0.370
SD	±0.004	±0.048	±0.130	±0.005	±0.005	±0.048

Tetracycline (3), diclofenac (4), ibuprofen (5), 17*α*-ethynylestradiol (6), nimesulide (8) ciprofloxacin (9), and norfloxacin (10).

**Table 4:** Analyte determination by the proposed and conventional methods.

Sample		Proposed method	Conventional methods
	Analyte	Concentration	Concentration
Moscados stream (*μ*g·L^−1^)	Caf	0.109 ± 0.002	0.086 ± 0.011
Cleópatra stream (*μ*g·L^−1^)	Caf	1.204 ± 0.034	1.358 ± 0.020
	Tetr	0.188 ± 0.007	0.195 ± 0.009
	Norflo	0.463 ± 0.022	0.427 ± 0.038
Borba Gato stream (*μ*g·L^−1^)	Caf	0.140 ± 0.005	0.136 ± 0.002
Mandacarú stream (*μ*g·L^−1^)	Caf	0.742 ± 0.024	0.766 ± 0.038
	Norflo	0.380 ± 0.016	0.395 ± 0.011
Morangueiro stream (*μ*g·L^−1^)	Caf	0.367 ± 0.020	0.371 ± 0.014
	Cipro	0.295 ± 0.010	0.281 ± 0.019
Maringá stream (*μ*g·L^−1^)	Caf	0.173 ± 0.010	0.190 ± 0.012
Potable water (*μ*g·L^−1^)	Caf	0.071 ± 0.003	0.064 ± 0.007

The conventional methods used were as follows: tetracycline [18]; ibuprofen, diclofenac, and nimesulide [24]; levonorgestrel and 17*α*-ethynylestradiol [25]; caffeine [29]; ciprofloxacin and norfloxacin [30]; amoxicillin [31].

**Table 5:** Recovery tests for water samples.

		Proposed method			Conventional methods		
Sample	Analyte	Recovery (%)	Precision (RSD, %)		Recovery (%)	Precision (RSD, %)	
		(*n*=3)	Intraday (*n*=3)	Interday (*n*=5)	(*n*=5)	Intraday (*n*=3)	Interday (*n*=5)
Moscados stream	Caf	99.52	0.94	1.53	98.39	1.21	1.67
	Tetra	98.64	1.33	2.89	96.67	2.04	2.55
	Cipro	98.92	2.45	3.90	96.20	2.83	4.81
	Norflo	99.14	1.09	1.47	98.80	1.55	2.39
	Diclo	99.45	1.77	0.95	99.94	0.90	1.96
	Ibup	98.20	1.61	2.11	98.85	1.32	1.42
	Nime	98.35	2.83	4.54	98.97	2.29	3.11
	Ethynyl	97.21	0.97	3.02	99.91	1.48	2.27
	Norg	99.08	3.14	4.67	99.87	2.11	2.89
	Amox	—	—	—	97.35	1.76	1.53

Cleópatra stream	Caf	97.85	2.37	3.10	98.78	1.05	2.14
	Tetra	99.71	0.59	2.48	99.89	1.49	2.95
	Cipro	98.22	0.83	1.26	98.96	1.68	3.28
	Norflo	101.30	1.11	1.45	100.13	2.27	1.99
	Diclo	99.45	0.76	1.90	98.32	1.84	2.56
	Ibup	99.60	1.28	2.34	98.88	1.16	1.59
	Nime	98.50	0.67	1.19	97.83	2.08	2.31
	Ethynyl	99.31	1.04	1.72	98.36	1.53	2.47
	Norg	98.06	0.95	1.58	98.97	1.87	2.08
	Amox	—	—	—	98.60	1.29	1.84

Borba Gato stream	Caf	98.23	0.61	1.04	98.52	2.08	3.77
	Tetra	99.11	1.58	3.27	99.01	1.73	2.01
	Cipro	97.89	1.13	1.85	99.27	0.99	1.63
	Norflo	98.04	1.20	2.01	99.17	1.62	1.94
	Diclo	99.55	0.44	1.33	98.05	1.31	2.22
	Ibup	99.19	0.82	1.59	98.82	1.47	1.98
	Nime	100.73	0.57	1.98	99.51	0.84	1.72
	Ethynyl	99.82	1.28	2.10	98.46	1.10	2.36
	Norg	98.44	0.49	1.67	99.04	1.26	1.93
	Amox	—	—	—	98.63	0.63	1.69

Mandacarú stream	Caf	100.91	1.23	1.81	98.39	0.55	1.14
	Tetra	97.36	1.05	2.17	98.67	1.62	2.51
	Cipro	99.94	1.41	2.32	97.20	1.28	1.93
	Norflo	99.12	1.95	3.06	98.80	1.99	2.48
	Diclo	101.33	1.03	1.90	99.94	2.11	2.83
	Ibup	99.60	0.55	1.26	98.85	1.33	1.70
	Nime	99.82	0.81	1.78	97.77	0.81	1.66
	Ethynyl	99.01	0.90	1.63	99.91	1.55	3.90
	Norg	100.29	0.78	1.99	99.87	0.90	1.93
	Amox	—	—	—	99.35	1.22	2.07

Morangueiro stream	Caf	99.14	0.32	0.98	98.78	0.95	1.57
	Tetra	98.52	0.70	1.53	99.89	0.49	1.00
	Cipro	98.05	1.51	2.49	98.96	1.35	2.41
	Norflo	98.81	1.63	2.72	100.13	1.21	2.27
	Diclo	99.20	0.76	1.44	98.32	0.88	1.82
	Ibup	100.33	0.52	0.97	98.88	2.04	2.89
	Nime	98.87	1.11	2.06	97.83	1.59	2.70
	Ethynyl	99.00	0.83	1.88	98.33	1.11	1.40
	Norg	100.94	1.47	2.91	98.97	0.97	1.34
	Amox	—	—	—	99.04	0.83	0.90

Maringá stream	Caf	97.99	1.14	2.03	98.52	0.66	1.32
	Tetra	99.10	0.95	1.82	99.01	1.01	1.83
	Cipro	97.66	1.98	3.06	98.27	1.44	1.68
	Norflo	98.34	1.32	2.21	99.17	1.27	2.83
	Diclo	99.62	1.77	3.48	99.05	0.59	1.16
	Ibup	98.41	0.92	1.85	98.82	1.33	2.29
	Nime	99.97	1.06	1.95	98.51	0.78	2.04
	Ethynyl	98.55	1.20	1.79	98.46	1.11	1.98
	Norg	99.67	1.00	1.53	99.04	1.50	2.10
	Amox	—	—	—	98.63	0.95	1.93

Tap water	Caf	99.96	0.25	0.81	100.40	2.05	1.19
	Tetra	99.05	2.05	2.97	98.00	1.39	1.72
	Cipro	99.31	0.68	1.14	97.93	1.03	1.58
	Norflo	99.20	0.92	1.67	98.33	0.76	1.47
	Diclo	100.08	0.74	1.93	99.19	0.62	0.95
	Ibup	99.19	0.33	0.85	100.71	1.28	1.33
	Nime	99.21	0.49	1.36	99.67	0.67	1.59
	Ethynyl	99.89	0.97	2.02	97.59	1.04	1.45
	Norg	98.40	0.81	1.77	97.71	0.95	1.90
	Amox	99.12	1.02	1.84	98.26	1.32	2.34

Data were obtained for water samples spiked with 5.0 *µ*g·L^−1^ of ibuprofen, 17*α*-ethynylestradiol, diclofenac, nimesulide, levonorgestrel, ciprofloxacin, and norfloxacin; 10 *µ*g·L^−1^ of caffeine; and 60 *µ*g·L^−1^ of amoxicillin. The conventional methods were the same as used in Table 4.
